# Self-Reported Changes in Exercise-Related Hydration Practices over a 30-Day Period in Physically Active University Students

**DOI:** 10.3390/sports14090381

**Published:** 2026-09-01

**Authors:** Danijela Kuna, Jasenka Gajdoš Kljusurić, Vesna Bosanac

**Affiliations:** 1 Faculty of Kinesiology Osijek, Josip Juraj Strossmayer University of Osijek, Drinska 16a, 31000 Osijek, Croatia; dkuna@kifos.hr; 2 Faculty of Food Technology and Biotechnology, University of Zagreb, Pierottijeva 6, 10000 Zagreb, Croatia; 3 Sports Nutrition, Lukaveča 29, 10412 Donja Lomnica, Croatia; vesna@prehranasportasa.com

**Keywords:** sports nutrition, beverage consumption, hydration practices, exercise behavior, temporal stability, repeated measures

## Abstract

Beverage consumption practices before, during, and after training are relevant to physical performance and health. However, less is known about whether these self-reported practices remain stable or change over relatively short periods. The primary aim of this study was to determine whether the frequency of self-reported exercise-related beverage consumption changed over a 30-day period in physically active university students. A secondary aim was to characterize individual-level temporal agreement in selected beverage consumption frequency items across the two assessments. A total of 34 participants (18 males and 16 females; age 20.8 ± 0.5 years) completed a 28-item questionnaire on two occasions separated by 30 days. Paired Wilcoxon signed-rank tests were used to examine group-level changes in ordinal beverage-consumption frequency, while weighted Cohen’s kappa coefficients (κw) with 95% confidence intervals, exact agreement, and adjacent-category agreement were used to characterize individual-level temporal agreement. No statistically significant group-level differences were detected between assessments for the analyzed beverage-consumption frequency items (all *p* > 0.05). Across the analyzed items, exact agreement ranged from 73.5% to 100.0%, adjacent-category agreement from 94.1% to 100.0%, and κw values ranged from 0.329 to 1.000. Water was the most frequently reported beverage before, during, and after training. Overall, no systematic group-level changes in exercise-related beverage consumption frequency were detected over the 30-day period, while individual-level temporal agreement varied across beverage categories and exercise-related time points.

## 1. Introduction

Adequate hydration is a fundamental component of health and athletic performance. Fluid balance plays a critical role in thermoregulation, cardiovascular function, and metabolic processes during physical activity [1]. Even mild dehydration can negatively affect endurance, strength, cognitive performance, and overall exercise capacity [1,2,3]. Furthermore, modest hypohydration of around 2–3% of body mass can increase cardiovascular and thermal strain and negatively affect technical skills and high-intensity efforts, especially in the heat [3,4]. Symptoms of dehydration, such as fatigue, increased perceived exertion, and discomfort, can also compromise cognitive function and sport-specific decision-making, particularly at higher levels of fluid loss and under heat stress [1,3,5]. Sweat rates and sweat sodium concentrations exhibit substantial inter-individual variability and are strongly influenced by exercise intensity, clothing or equipment, and environmental conditions, making generalized “one-size-fits-all” hydration recommendations suboptimal [2,5,6,7].

Athletes are particularly vulnerable to fluid imbalances due to increased sweat rates, environmental conditions, and the intensity and duration of training [7,8]. For this reason, appropriate hydration strategies, including the timing, quantity, and composition of fluid intake, are essential for maintaining optimal performance, preventing dehydration-related complications, and avoiding overhydration and exercise-associated hyponatremia [2,6].

Although guidance is available for fluid intake before, during, and after exercise, no single daily fluid-intake prescription is appropriate for all athletes or physically active individuals. Exercise-related hydration strategies should therefore be individualized according to the characteristics of the individual, the type, intensity, and duration of exercise, sweat losses, environmental exposure, opportunities to drink, and the time available for recovery [6,9].

Nevertheless, previous studies suggest that hydration practices among athletes are often inconsistent and influenced by individual habits, level of education, and access to professional guidance [10,11,12]. Knowledge, attitudes, and practices regarding fluid replacement are frequently suboptimal, with many athletes relying on thirst alone, failing to hydrate before exercise, or misunderstanding the risks of both hypohydration and hyperhydration. While water remains the primary source of hydration [1,8,10], the use of electrolyte-containing beverages and other specialized drinks varies considerably across different athletic populations, partly reflecting sport type, environmental demands, and perceived performance benefits [13,14,15].

Hydration-related behaviors can be assessed using prospective fluid records, dietary recalls, beverage-frequency questionnaires, and structured self-report questionnaires [16,17,18,19]. Objective measures, such as exercise-related body mass changes, urine specific gravity, and urine osmolality, can provide information about hydration status but do not necessarily describe habitual beverage selection or the timing of fluid intake [1,17,19]. Self-administered questionnaires offer a practical and economical method for assessing hydration-related behaviors in larger groups. Repeated assessment of these practices can provide information on their short-term stability and variation over time.

Although exercise-related hydration behaviors have been described in different athletic and physically active populations, relatively little is known about their short-term temporal stability within the same individuals. Such behaviors may reflect habitual practices, but they can also vary according to changes in training load, exercise characteristics, environmental conditions, dietary behavior, and other contextual factors. Repeated assessment over a defined short-term period can therefore provide information on whether self-reported exercise-related hydration practices remain broadly stable or show measurable changes over time.

The choice of the interval between repeated assessments is important when examining short-term behavioral patterns. An interval that is too short may increase the possibility that participants remember their previous responses and reproduce them, whereas a substantially longer interval increases the opportunity for genuine changes in behavior and contextual conditions. Previous research involving physically active individuals has used a one-month interval between repeated administrations of dietary and training-related questionnaires, and one-month intervals have also been used in more recent dietary assessment studies in athletes [18,20,21]. In addition, a systematic review of dietary assessment in athletes has highlighted the importance of considering the short-term variability associated with training microcycles and periodization when repeated dietary assessments are interpreted. This is supported by research on physically active individuals, which repeated the questionnaire after one month [22], as well as recent work on athletes, which also explicitly uses one month for reproducibility [23].

Accordingly, a 30-day interval was selected in the present study as a pragmatic short-term period that was long enough to reduce the likelihood of immediate recall of the first questionnaire responses, while remaining sufficiently short to examine temporal stability and potential changes in exercise-related hydration practices. Importantly, this interval does not assume that participants’ behaviors remained unchanged. Rather, differences observed between assessments may reflect genuine short-term changes in hydration practices or variation in self-reported responses, and these possibilities should be considered when interpreting the findings.

The primary aim of this study was therefore to determine whether the frequency of self-reported exercise-related beverage consumption changed over a 30-day period in physically active university students. A secondary aim was to characterize individual-level temporal agreement in selected beverage consumption frequency items across the two assessments [17,18,19,24].

## 2. Materials and Methods

### 2.1. Participants

A total of 34 physically active university students (18 males and 16 females) were included in the study. Participants were students at the Faculty of Kinesiology, University of Split, Croatia, with a mean age of 20.8 ± 0.5 years. Participants were recruited using a convenience sampling approach from undergraduate cohorts enrolled in sport-related study programs. Participants classified as noncompetitive regularly participated in organized sport or structured exercise training but did not participate in formal sport competitions. Participants reported involvement in kickboxing, athletics, football, baseball, taekwondo, weightlifting, gym- and fitness-based exercise, and various recreational sports activities. Inclusion criteria were regular participation in organized sports training and the absence of medical conditions affecting hydration status. Exclusion criteria included recent injury, illness, or any condition that could influence habitual fluid intake. A total of 40 individuals were initially contacted. Of these, four did not complete participation, resulting in 36 participants who completed the first questionnaire administration. During the second administration, two questionnaires were incomplete and were therefore excluded from the analysis. Consequently, 34 participants provided complete paired responses and constituted the final analytical sample. No a priori sample-size calculation was performed. All participants with complete paired responses from both assessments were included in the analyses. Participation was voluntary, and all participants provided written informed consent prior to inclusion in the study. The study protocol was reviewed and approved by the Institutional Ethics Committee of the Faculty of Kinesiology, University of Zagreb, Croatia (Approval No. 101/2016). All procedures were conducted in accordance with the Declaration of Helsinki.

### 2.2. Study Design and Procedure

A repeated-measures observational design was used to examine short-term stability and change in self-reported exercise-related beverage consumption practices. The same structured questionnaire was administered on two occasions separated by exactly 30 days. The first questionnaire administration was conducted in March 2018, and the second was conducted 30 days later, in April 2018. A 30-day interval was selected because intervals of approximately one month have been used in previous repeated dietary and hydration-related assessments [18,20,21,22,23], while providing a sufficiently long period to reduce the likelihood of immediate recall of responses from the first administration. At the same time, the interval was intended to represent a short-term period in which exercise-related hydration practices could be examined for stability or change rather than assuming that behavior remained constant. All assessments were conducted under standardized conditions during morning classes, prior to physical activity sessions, to minimize the influence of acute exercise on participants’ responses. The questionnaire was self-administered, and no intervention or educational input was provided between the assessments, thereby reducing the likelihood that differences between assessments were attributable to a study-induced change. Participants were instructed to complete the questionnaire independently and without discussing their responses with others, while a researcher was present to clarify procedural questions if needed. Anthropometric and background data were collected at both assessments. Body mass and height were self-reported by the participants, and body mass index (BMI) was calculated as body mass in kilograms divided by height in meters squared (kg/m^2^).

### 2.3. Self-Administered Questionnaire

The questionnaire was developed and pilot-tested by the same research group in a separate sample of 17 university students [24]. In that study, the reproducibility of questions addressing sport participation and hydration practices before, during, and after training was examined using correlation coefficients, corrected Cohen’s d values, and chi-square tests. Following the pilot evaluation, items with lower reproducibility were revised, and the resulting final version was administered to the separate sample of 34 participants included in the present study. The questionnaire was administered in Croatian and consisted of 28 questions organized into five sections: (1) demographic, self-reported anthropometric, and sport-related characteristics; (2) hydration practices before training; (3) hydration practices during training; (4) hydration practices after training; and (5) general hydration practices and sources of hydration-related information.

The questionnaire assessed the frequency, timing, usual volume, and temperature of beverage consumption before, during, and after training. It also assessed the reasons for beverage consumption, seasonal changes in fluid intake, participants’ self-assessment of their overall fluid intake, and the frequency and sources of hydration-related information. A total of 10 predefined beverage categories were included, representing the most commonly consumed drinks among athletes, such as water, electrolyte drinks, protein beverages, and energy drinks. An open-ended “other beverage” option was also provided.

The selection of beverage categories was based on their relevance to hydration behaviors and their prevalence in athletic populations. Beverage-consumption frequency was assessed using four ordinal response categories: “every time”, “occasionally”, “rarely”, and “never”. Beverage volume was also assessed using predefined ordinal categories, ranging from non-consumption to more than 500 mL. Non-consumption was indicated by “never” for frequency items and by “I do not consume this fluid” for volume items. Participants responded separately for each beverage category and could therefore report the consumption of multiple beverage types. Multiple responses were permitted for questions concerning beverage temperature. The complete wording and response options for all questions are provided in the Supplementary Materials.

**Figure 1:**
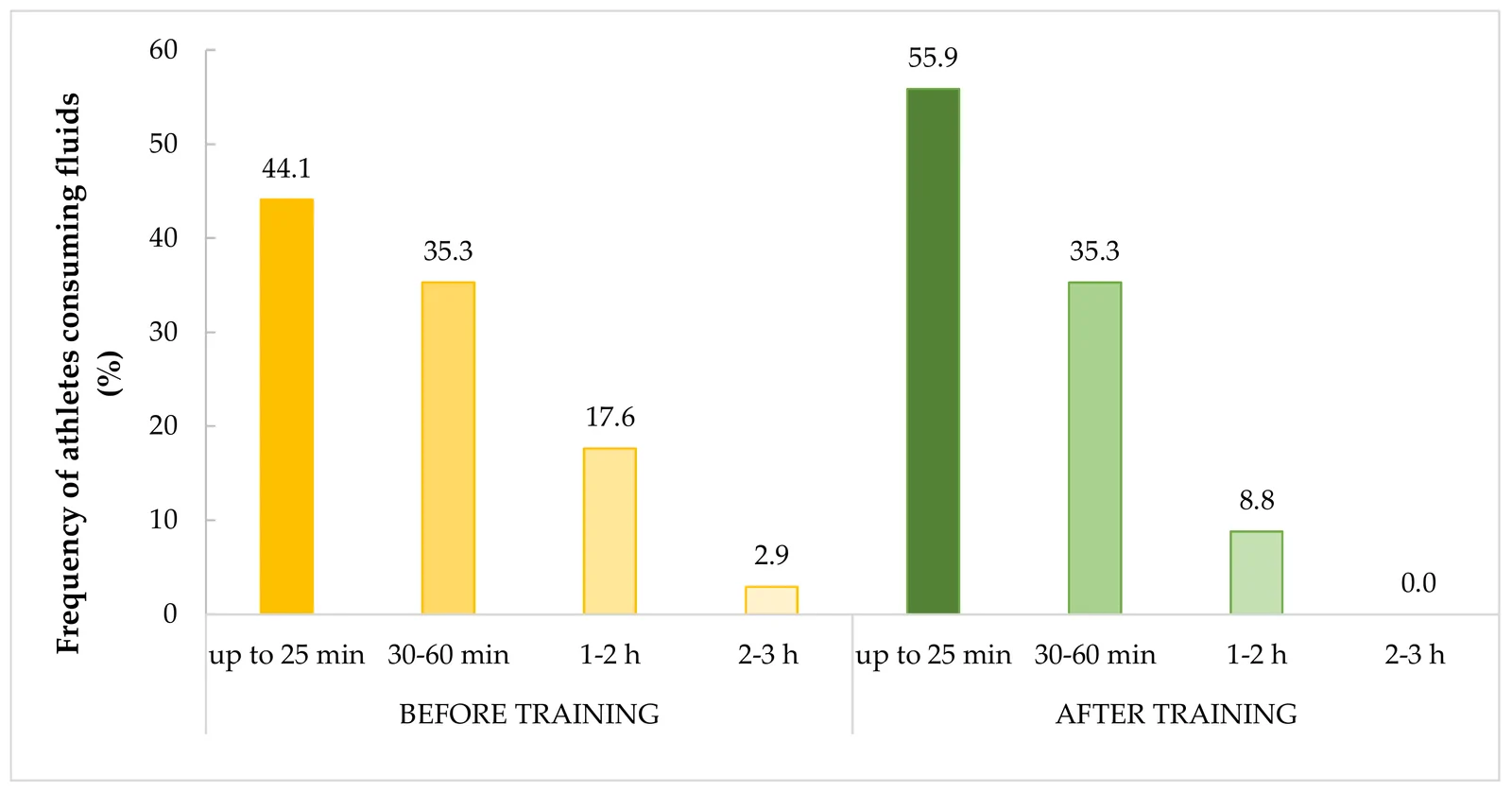
Timing of greatest beverage intake before and after training at the first assessment.

Participants were instructed to report their usual exercise-related hydration practices rather than intake during a specified retrospective period. This approach was consistent with the purpose of the questionnaire, which was designed to characterize habitual beverage-consumption practices before, during, and after training rather than to quantify total daily fluid intake over a defined number of days. Because training and environmental conditions may vary, participants were asked to consider their usual practices across their ongoing training routine. The absence of a fixed retrospective recall period should nevertheless be considered when interpreting the findings, as participants may have applied somewhat different reference periods when describing their usual practices at the two assessments. The final section assessed sources of information regarding hydration practices. Responses were analyzed separately at the item level, and no overall questionnaire score was calculated. Ordinal response categories were retained in their natural order for the paired analyses between the two assessments. The complete original Croatian-language questionnaire and its English translation prepared for publication purposes are provided as Supplementary Files S1 and S2, respectively. The primary analysis focused on paired responses to selected ordinal items assessing beverage consumption frequency before, during, and after training across the two assessments. The remaining questionnaire items were used for participant characterization or descriptive presentation and were not included in the paired analysis across the two assessments. The questionnaire items assessing the timing of greatest fluid intake before and after training (Questions 14 and 23) were retained in the present study because timing is an important component of exercise-related hydration practices. These items were included in the descriptive analysis of the first questionnaire administration and are presented in Figure 1. They were not included in the primary paired analysis because that analysis was predefined to focus on the selected ordinal beverage-consumption frequency items administered across the three exercise-related time points (before, during, and after training). Accordingly, Questions 14 and 23 were used to provide contextual information about hydration timing, whereas the longitudinal statistical analysis was restricted to the selected frequency items for which the same ordinal response structure was available across the two assessments. The use of the questionnaire items in the present study is summarized in Table S1.

### 2.4. Data Analysis

Statistical analyses were conducted using Python (version 3.10; scikit-learn and SciPy packages) and R (version 4.2). Descriptive statistics are presented as mean ± standard deviation (SD) for continuous variables and as frequencies and percentages for categorical variables. Paired Wilcoxon signed-rank tests were used to examine systematic differences in ordinal beverage consumption frequency between the two assessments.

To characterize individual-level temporal agreement between assessments, linear weighted Cohen’s kappa coefficients (κw) with 95% confidence intervals (95% CIs) were calculated for the selected ordinal beverage consumption frequency items. Exact agreement was calculated as the percentage of participants who selected the same response category on both occasions, whereas adjacent-category agreement represented the percentage of responses that were identical or differed by no more than one ordinal category between the two assessments.

Weighted kappa coefficients were interpreted according to the criteria proposed by Landis and Koch [25] as follows: ≤0.20, slight agreement; 0.21–0.40, fair agreement; 0.41–0.60, moderate agreement; 0.61–0.80, substantial agreement; and 0.81–1.00, almost perfect agreement. Spearman’s rank correlation coefficients (ρ) were additionally calculated to assess the consistency of participants’ rank ordering between assessments.

Only beverage categories with available paired responses from both assessments were included in the analyses. Sweetened coffee, sweetened tea, and other beverages were excluded because paired responses were unavailable. For the Wilcoxon signed-rank tests, statistical significance was set at *p* < 0.05.

## 3. Results

The baseline characteristics of the study participants are presented in Table 1, while the basic anthropometric data are presented in Table 2. A total of 34 participants (52.9% male and 47.1% female) were included in the study, with a mean age of 20.8 ± 0.5 years (Table 1). The majority of participants did not participate in formal sport competitions (64.7%), while 20.6% competed at the national and international levels as members of a national team. Most participants reported engaging in 3–7 training sessions per week.

As shown in Table 2, anthropometric characteristics and weekly training duration were descriptively similar across the two assessments.

### 3.1. Beverage Consumption Frequency Across Repeated Assessments

Results for beverage consumption frequency across the two assessments, including group-level changes and temporal agreement, are presented in Table 3.

No statistically significant group-level differences in beverage consumption frequency were detected between the two assessments for any of the analyzed beverage categories before, during, or after training (all *p* > 0.05). At the individual level, temporal agreement varied across beverage categories and exercise-related time points. Exact agreement ranged from 73.5% to 100.0%, while adjacent-category agreement ranged from 94.1% to 100.0%. Water showed substantial agreement across all three exercise-related time points (κw = 0.71–0.79), while electrolyte beverages and protein drinks also showed predominantly substantial agreement (κw = 0.68–0.82 and κw = 0.76–0.80, respectively). Weighted kappa coefficients for the remaining beverage categories ranged from 0.329 to 1.000. For several infrequently reported beverage categories, responses were identical across the two assessments. However, these values occurred in the context of highly concentrated response distributions.

### 3.2. Beverage Consumption Practices

Table 4 presents descriptive data on the self-reported amounts of beverages consumed before, during, and after training at the first assessment. Water was reported in the greatest amounts across all three time points. Before training, the most frequently reported amount of water was 300–500 mL, whereas during and after training, participants most commonly reported consuming either 300–500 mL or more than 500 mL.

Other beverage categories were generally reported in smaller amounts. Protein drinks showed a somewhat different pattern and were reported mainly before and after training, most commonly in the 100–200 mL category.

As shown in Figure 1, 44.1% of participants reported consuming the greatest amount of fluid within 25 min before training, whereas 55.9% reported consuming the greatest amount within 25 min after training. These findings complement the beverage-frequency results by showing that exercise-related hydration practices also included a distinct temporal component, with participants reporting the greatest fluid intake predominantly within 25 min before or after training.

**Figure 2:**
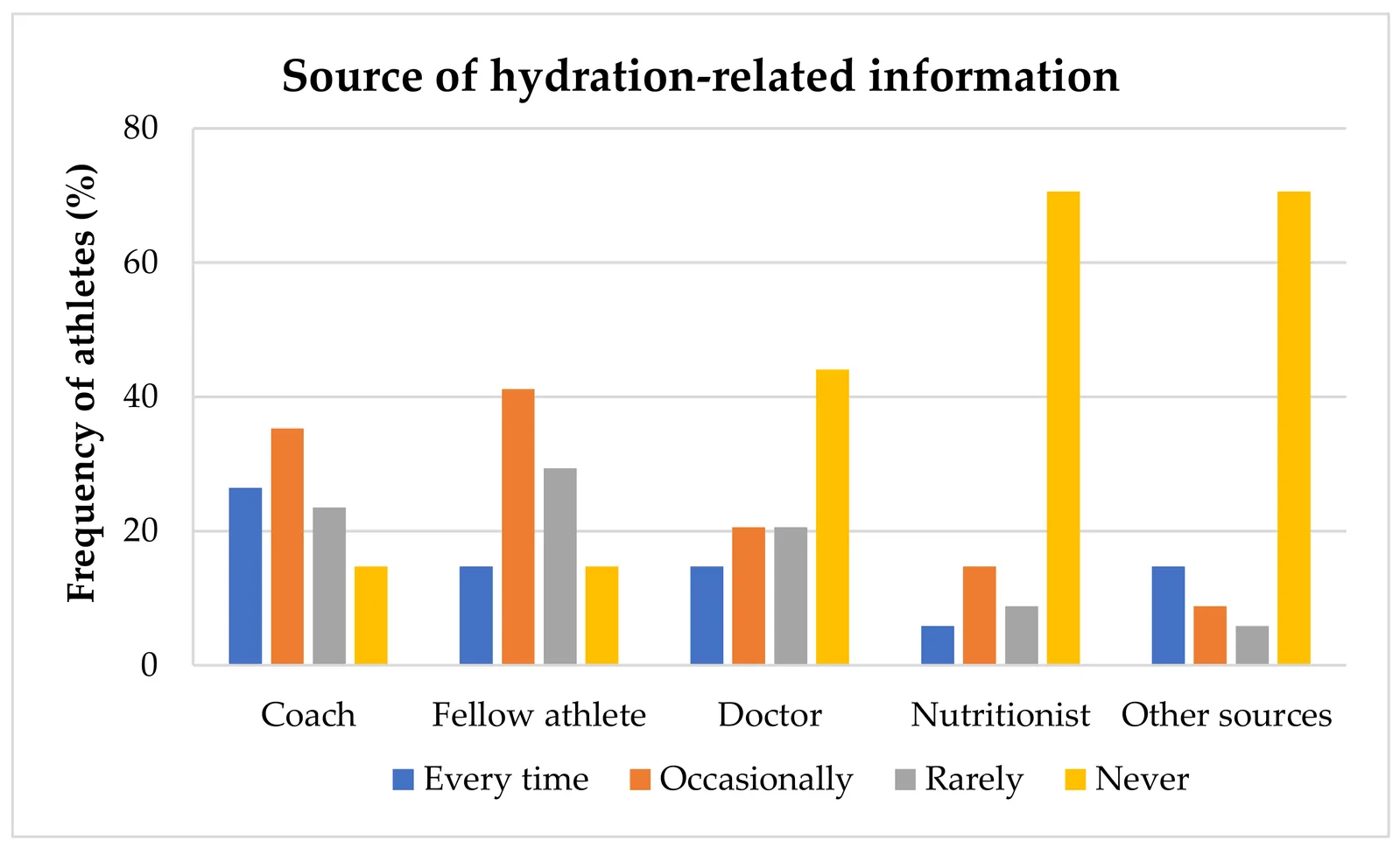
Frequency of obtaining hydration-related information from different sources at the first questionnaire administration. Coaches were the most frequently reported source of hydration-related information, followed by fellow athletes.

As shown in Figure 2, coaches were the most frequently reported source of hydration-related information, followed by fellow athletes.

## 4. Discussion

The main finding of this study was that no statistically significant group-level changes in self-reported beverage consumption frequency were detected over the 30-day period. At the individual level, however, temporal agreement varied across beverage categories and exercise-related time points. The most consistent findings were observed for water, electrolyte beverages, and protein drinks. Weighted kappa coefficients for water ranged from 0.71 to 0.79 across the periods before, during, and after training, while the corresponding ranges were 0.68–0.82 for electrolyte beverages and 0.76–0.80 for protein drinks. Across all analyzed items, exact agreement ranged from 73.5% to 100.0%, whereas adjacent agreement ranged from 94.1% to 100.0%. Temporal agreement was not equally strong across all beverage categories. Weighted kappa coefficients for fruit juice ranged from 0.497 to 0.579, while the lowest coefficient was observed for liquid carbohydrate drinks after training (κw = 0.329; 95% CI [−0.104, 0.790]). For this item, exact agreement was nevertheless 82.4%, and adjacent agreement was 94.1%. The high adjacent-category agreement indicates that most discrepancies involved a shift of no more than one ordinal response category. This combination illustrates why weighted kappa coefficients should be considered together with observed agreement and confidence intervals, particularly when responses are unevenly distributed across ordinal categories [26]. Several infrequently reported beverage categories yielded κw values of 1.00, including classic cola drinks during and after training, energy drinks after training, amino acids before training, and liquid carbohydrate drinks during training. Although these values indicate identical responses across the two assessments, they do not necessarily identify the beverage categories with the greatest temporal stability. When most participants select the same response category, perfect agreement may primarily reflect limited variability in responses. The wide confidence intervals obtained for several other infrequently reported beverages further demonstrate the limited precision of some item-level estimates in this relatively small sample [26]. An important contribution of the present study is the combined presentation of chance-corrected agreement, raw agreement percentages, and 95% confidence intervals at the item level. This approach provides a more complete description of individual-level temporal agreement across the two assessments than relying exclusively on correlations or tests of systematic differences. Wilcoxon signed-rank tests were used to examine systematic group-level changes between the two assessments, whereas Spearman’s rank correlation coefficients were used to describe rank-order consistency. A high Spearman coefficient indicates that participants retained a similar relative ranking across assessments, even when their absolute responses differed. Similarly, a nonsignificant Wilcoxon result indicates that no systematic group-level shift was detected but does not demonstrate that individual participants selected the same response category at both assessments. Agreement coefficients and their confidence intervals therefore provide complementary information on individual-level temporal agreement between assessments [27,28].

Previous studies have examined questionnaires assessing water intake, beverage habits, and hydration-related behaviors [17,18,19]. Direct numerical comparisons are difficult because these instruments differ in their purpose, structure, recall period, target population, and statistical procedures. Some questionnaires were designed to estimate habitual or total daily water intake or hydration status, whereas the present questionnaire focused on beverage-consumption practices in relation to training. The questionnaire used in the present study was previously developed and pilot-tested by the same research group [24], while the present study examined selected beverage-consumption frequency items across two assessments in a separate sample. Accordingly, direct comparisons should account for differences in what the respective questionnaires were designed to assess.

Water was the most commonly reported beverage and was consumed in the greatest reported amounts before, during, and after training at the first assessment. In the repeated assessment of beverage consumption frequency, water also showed substantial temporal agreement across all three exercise-related time points (κw = 0.71–0.79), suggesting that this practice was relatively consistent over the 30-day period. This pattern is consistent with the central role of water in replacing exercise-related fluid losses [1,6,29]. However, frequent water consumption and substantial temporal agreement between assessments do not establish that participants were adequately hydrated or that their exercise-related hydration practices corresponded to individual requirements. Appropriate exercise-related hydration requirements depend on exercise duration and intensity, sweat rate, sweat sodium concentration, environmental conditions, acclimatization status, clothing and equipment, and opportunities to drink [1,6,7,29]. As these factors and objective hydration indicators were not assessed, the adequacy of participants’ hydration practices could not be determined.

In contrast to water, other specialized beverages were reported less frequently. Their lower use may reflect differences in training demands, individual preferences, or the perceived need for these products; however, these factors were not assessed in the present study. Carbohydrate-containing beverages may support performance during prolonged exercise under specific conditions [30]. However, the relevance of specialized beverages depends on the characteristics of the exercise and the individual requirements of the athlete [2,6]. The present study did not document session duration and intensity, sweat losses, dietary intake, or individual energy and electrolyte requirements. It is therefore not possible to determine whether the limited consumption of these products represented an appropriate choice or an unmet nutritional or hydration need. Protein drinks were reported more frequently before and after training than several other non-water beverages. This pattern may reflect their association with recovery-related nutrition practices among physically active students. However, the questionnaire did not assess total dietary protein intake, energy intake, training goals, or whether supplementation was indicated for individual participants. The findings consequently describe the frequency and timing of protein-drink consumption but do not provide evidence regarding its necessity, effectiveness, or appropriateness.

Coaches were the most frequently reported source of hydration-related information, followed by fellow athletes. This finding is consistent with previous research indicating that coaches may play an important role in shaping nutrition- and hydration-related behaviors [12]. From a practical perspective, it emphasizes the importance of ensuring that information communicated within training environments is evidence-based and appropriately individualized. However, the present study did not assess the accuracy, quality, or specific content of the advice received from these sources.

Several limitations should be acknowledged. The major limitation of the present study is the 30-day interval between questionnaire administrations and the possibility that participants’ exercise-related hydration practices changed during this period. Training load, exercise type and frequency, dietary behavior, environmental exposure, health status, and other contextual factors were not systematically monitored throughout the 30-day interval. Consequently, differences between the two assessments cannot be attributed exclusively to variation in self-reporting and may partly reflect genuine changes in participants’ hydration practices. Conversely, the absence of a statistically significant group-level change does not demonstrate that individual behaviors remained unchanged. The 30-day interval was selected as a pragmatic short-term period for examining temporal stability and change, but it should not be interpreted as a period during which participants’ behaviors were expected to remain constant. A second limitation concerns the absence of a fixed retrospective recall period. Participants were instructed to report their usual exercise-related hydration practices, which was consistent with the intended assessment of habitual behavior but may have allowed participants to use somewhat different reference periods at the two assessments. This may have contributed to variation in responses independently of actual changes in hydration behavior. The study also included a relatively small convenience sample of 34 physically active university students with a narrow age range, and most participants did not participate in formal sport competitions. The findings may therefore not be generalizable to elite athletes, athletes from particular sports, younger or older populations, or individuals exposed to substantially different training demands.

All questionnaire data were self-reported and may have been affected by recall difficulties, social desirability, or differences in the interpretation of ordinal response categories. As noted above, no fixed retrospective recall period was specified. Instead, participants were instructed to describe their usual hydration practices. Although this approach was intended to capture habitual behavior, the meaning of “usual practices” may have been interpreted differently across the two assessments. In addition, no objective indicators of hydration status or beverage intake, such as urine indices, prospectively recorded fluid intake, sweat loss, or pre- to post-exercise body-mass changes, were collected. Different hydration indices may also vary in their ability to detect exercise-induced dehydration [31]. The study therefore cannot establish whether the reported practices corresponded to actual beverage intake or physiological hydration status.

Several beverage categories had highly concentrated response distributions, resulting in perfect kappa coefficients for some items and wide confidence intervals for others. These estimates should therefore be considered in conjunction with exact and adjacent agreement rather than interpreted in isolation. In addition, the present agreement analysis focused on beverage-consumption frequency items. Accordingly, the findings regarding temporal agreement apply specifically to these frequency items and cannot be extended to beverage amount, temperature, timing, sources of information, or the questionnaire as a whole.

Future studies should examine short-term changes in exercise-related beverage consumption practices in larger and more heterogeneous samples representing different sports, competitive levels, age groups, and environmental settings. Repeated assessments across different seasons and training phases would also be valuable, particularly when changes in training load, dietary behavior, health status, and environmental exposure are documented throughout the assessment period. Comparisons with prospectively recorded fluid intake and objective hydration measures would help determine whether self-reported practices accurately reflect participants’ actual behavior and physiological hydration status.

## 5. Conclusions

Because contextual factors were not systematically monitored during the 30-day interval, the findings should be interpreted as evidence of observed temporal stability at the group level rather than proof that individual hydration behaviors remained unchanged. At the first assessment, water was the most commonly reported beverage and was consumed in the greatest reported amounts before, during, and after training. These findings describe self-reported exercise-related beverage practices and should not be interpreted as an assessment of total daily fluid intake, hydration adequacy, or physiological hydration status.

## Figures and Tables

**Table 1:** Baseline characteristics of participants.

Variable	Category	*n*	%
Sex	Male	18	52.9
	Female	16	47.1
Competitive status	Non-competitive	22	64.7
	Regional level	0	0.0
	National level	1	2.9
	National/international level, not national team member	4	11.8
	National/international level, national team member	7	20.6
	Olympian	0	0.0
Training frequency	3–4 sessions/week	12	35.3
	5–7 sessions/week	14	41.2
	8–10 sessions/week	6	17.6
	11–14 sessions/week	2	5.9

**Table 2:** Anthropometric characteristics of participants at two assessments.

Second Assessment			First Assessment			Observed Parameters
All	F	M	All	F	M	
21.0 ± 0.3	21.0 ± 0.0	21.0 ± 0.5	20.8 ± 0.5	20.9 ± 0.4	20.8 ± 0.7	Age (years)
73.3 ± 13.3	62.3 ± 7.0	83.1 ± 8.9	72.8 ± 12.9	62.1 ± 7.2	82.3 ± 8.5	Body mass (kg)
178.8 ± 8.7	172.3 ± 6.3	184.6 ± 6.2	178.9 ± 8.8	172.3 ± 6.3	184.8 ± 6.1	Body height (cm)
22.8 ± 2.4	20.9 ± 1.7	24.4 ± 1.8	22.6 ± 2.3	20.9 ± 1.8	24.1 ± 1.5	BMI (kg/m^2^)
2.9 ± 1.3	2.9 ± 1.1	2.9 ± 1.5	2.8 ± 1.1	2.5 ± 0.9	3.11 ± 1.2	Training duration score

Note. Values are presented as mean ± SD. M, males; F, females; BMI, body mass index. The training duration score was coded on a five-point scale ranging from 1 (4–5 h/week) to 5 (≥17 h/week). Physical activities that were an integral part of the participants’ study programme were not included.

**Table 3:** Assessment of stability and change in self-reported beverage consumption frequency over 30 days.

Beverage Item	Timing	Exact (%)	Adjacent (%)	κw	95% CI	ρ	Wilcoxon *p*
Water	Pre	76.5	97.1	0.740	[0.580, 0.900]	0.780	0.412
	During	79.4	100.0	0.790	[0.640, 0.940]	0.820	0.655
	Post	73.5	94.1	0.710	[0.530, 0.890]	0.750	0.317
Electrolyte beverages	Pre	88.2	100.0	0.680	[0.420, 0.940]	0.710	0.820
	During	94.1	100.0	0.820	[0.610, 1.000]	0.840	0.999
	Post	88.2	97.1	0.730	[0.500, 0.960]	0.760	0.564
Protein drinks	Pre	82.4	97.1	0.760	[0.570, 0.950]	0.790	0.480
	During	91.2	100.0	0.800	[0.590, 1.000]	0.820	0.710
	Post	76.5	97.1	0.770	[0.600, 0.940]	0.800	0.610
Fruit juice	Pre	82.4	94.1	0.579	[0.023, 1.000]	0.591	1.000
	During	82.4	100.0	0.523	[0.000, 0.790]	0.540	0.564
	Post	88.2	94.1	0.497	[0.047, 1.000]	0.763	0.655
Classic cola drinks	Pre	94.1	100.0	0.638	[0.000, 1.000]	0.685	0.317
	During	100.0	100.0	1.000	[1.000, 1.000]	1.000	1.000
	Post	100.0	100.0	1.000	[1.000, 1.000]	1.000	1.000
Energy drinks	Pre	76.5	94.1	0.612	[0.000, 0.930]	0.679	0.705
	During	88.2	100.0	0.717	[0.000, 1.000]	0.814	0.157
	Post	100.0	100.0	1.000	[1.000, 1.000]	1.000	1.000
Liquid carbohydrates	Pre	82.4	100.0	0.523	[0.000, 0.790]	0.540	0.564
	During	100.0	100.0	1.000	[1.000, 1.000]	1.000	1.000
	Post	82.4	94.1	0.329	[−0.104, 0.790]	0.430	1.000
Amino acids	Pre	100.0	100.0	1.000	[1.000, 1.000]	1.000	1.000
	During	88.2	100.0	0.779	[0.000, 1.000]	0.615	0.157
	Post	82.4	94.1	0.698	[0.084, 0.935]	0.808	0.414

Note. Pre = before training; During = during training; Post = after training; Exact = percentage of identical responses at both assessments; Adjacent = percentage of responses that were identical or differed by no more than one ordinal category; κw = linear weighted Cohen’s kappa; CI = confidence interval; ρ = Spearman’s rank correlation coefficient; Wilcoxon *p* = *p*-value from the Wilcoxon signed-rank test.

**Table 4:** Frequency of participants reporting each exercise-related beverage volume consumed before, during, and after training at the first assessment.

Proportion of Participants (%)					
Beverages	<100 mL	100–200 mL	200–300 mL	300–500 mL	>500 mL
	before training				
Water	2.9	11.8	26.5	38.2	20.6
Electrolytes	5.9	2.9	2.9	2.9	0.0
Fruit juice	5.9	2.9	5.9	2.9	0.0
Classic cola drinks	8.8	2.9	0.0	0.0	0.0
Sweetened coffee	8.8	5.9	0.0	0.0	0.0
Sweetened tea	8.8	8.8	0.0	0.0	0.0
Energy drinks	5.9	17.6	0.0	0.0	0.0
Protein drinks	0.0	23.5	8.8	2.9	0.0
Liquid carbs	2.9	8.8	0.0	0.0	0.0
Amino acids	0.0	20.6	0.0	2.9	0.0
Other beverages	8.8	2.9	0.0	0.0	0.0
	during training				
Water	2.9	8.8	20.6	35.3	32.4
Electrolytes	0.0	2.9	0.0	0.0	0.0
Fruit juice	2.9	5.9	0.0	0.0	0.0
Classic cola drinks	0.0	0.0	0.0	0.0	0.0
Sweetened coffee	0.0	0.0	0.0	0.0	0.0
Sweetened tea	0.0	0.0	0.0	0.0	0.0
Energy drinks	2.9	8.8	0.0	0.0	0.0
Protein drinks	2.9	8.8	0.0	0.0	0.0
Liquid carbs	2.9	0.0	0.0	0.0	0.0
Amino acids	5.9	0.0	0.0	0.0	0.0
Other beverages	0.0	0.0	0.0	0.0	0.0
	after training				
Water	5.9	14.7	20.6	26.5	32.4
Electrolytes	0.0	5.9	2.9	2.9	0.0
Fruit juice	2.9	5.9	0.0	2.9	0.0
Classic cola drinks	5.9	0.0	0.0	0.0	0.0
Sweetened coffee	5.9	2.9	0.0	0.0	0.0
Sweetened tea	5.9	2.9	0.0	0.0	0.0
Energy drinks	2.9	5.9	0.0	0.0	0.0
Protein drinks	5.9	20.6	17.6	8.8	0.0
Liquid carbs	0.0	2.9	0.0	2.9	0.0
Amino acids	5.9	2.9	2.9	0.0	0.0
Other beverages	0.0	0.0	0.0	0.0	0.0

Note. Percentages are based on *n* = 34 at the first assessment. Non-consumption responses are not shown, so row totals may be below 100%. Values represent self-reported beverage volumes before, during, and after training and do not represent total daily fluid intake. A value of 0.0 indicates that no consumption was reported in the corresponding category.

## Data Availability

The data presented in this study are available on request from the corresponding author.

## Supplementary Materials

The following supporting information can be downloaded at: https://www.mdpi.com/article/10.3390/sports14090381/s1, File S1, Original Croatian-language questionnaire; File S2, English translation of the questionnaire; Table S1, Questionnaire items and their use in the present study.
