# Supplementary material for: Self-Reported Changes in Exercise-Related Hydration Practices over a 30-Day Period in Physically Active University Students
Source: Sports (Basel). 2026 Sep 1;14(9):381. doi: 10.3390/sports14090381 (PMC13611659; doi:10.3390/sports14090381)
Supplement: Supplementary file 1 [file sports-14-00381-s001.zip › File_S1_Croatian_Questionnaire.pdf]

ANKETA O HIDRACIJI SPORTAŠA

Original Croatian-language questionnaire

Poštovane sportašice i sportaši,

U tijeku je znanstveno istraživanje koje ima cilj ispitati konzumaciju tekućine među sportašima koji se bave različitim sportskim aktivnostima. Anketni upitnik je anoniman, a namijenjen je sportašima i sportašicama starijim od 18 godina. Dobiveni će podaci biti korišteni isključivo u grupnom obliku za znanstvenu analizu. Za ispunjavanje ankete potrebno je oko 5 minuta.
Hvala na vašem trudu i sudjelovanju.

1. Navedite državu u kojoj živite. ______________________________

2. Odaberite klimu mjesta gdje uglavnom trenirate.

a) Tropska klima b) Suha klima c) Umjerena klima d) Snježno-šumska klima e) Polarna (ili snježna) klima

3. Spol a) Muškarac b) Žena

4. Dob ________________

5. Tjelesna visina (cm) ________________

6. Tjelesna masa (kg) __________________

7. Kako biste ocijenili svoje zdravstveno stanje?

a) loše b) zadovoljavajuće c) dobro d) vrlo dobro e) izvrsno

8. Kojim se sportom trenutno bavite? _________________________

9. Koliko godina se bavite tim sportom? _______________________

10. Odaberite tvrdnju koja Vas kao sportaša najbolje opisuje
a) Ne natječem se
b) Natječem se u regiji unutar države
c) Natječem se na nivou države
d) Natječem se u državi i inozemstvu no nisam član državne reprezentacije
e) Natječem se u državi i inozemstvu i član sam državne reprezentacije
f) Olimpijac

11. Odaberite prosječan broj treninga koji imate tijekom tjedna
a) 3–4 b) 5–7 c) 8–10 d) 11–14 e) 15 i više

12. Odaberite prosječan broj sati koliko trenirate tijekom tjedna
a) 4 - 5 b) 6–8 c) 9 - 12 d) 13–16 e) 17 i više

13. Na kojoj razini intenziteta dominiraju vaši treninzi?
a) od 30 do 40% intenziteta (niski) b) od 40 do 50% intenziteta (umjereni)

c) od 50 do 70% intenziteta (srednji) d) od 70 do 90% intenziteta (submaksimalni)
e) od 90% i više intenziteta (maksimalni)

14. U kojem vremenskom razdoblju PRIJE TRENINGA popijete najviše tekućine?
a) do 25 minuta prije treninga b) od 30 minuta do 1:00 sat prije treninga
c) od 1:00 do 2:00 sata prije treninga d) od 2:00 do 3:00 sata prije treninga

15. Označite učestalost uzimanja tekućina PRIJE TRENINGA

	Svaki put	Povremeno	Rijetko	Nikada
Voda				
Elektroliti				
Voćni sok				
Klasični cola napitci				
Zaslađena kava				
Zaslađeni čaj				
Energetski napitak (npr. Red Bull i sl.)				
Proteinski napitak				
Ugljikohidrati u tekućem obliku				
Aminokiseline				
Neki drugi napitak (ovdje odaberite učestalost, a vrstu navedite ispod)				
Vrsta_______________________				

16. Odaberite količinu tekućine koju uzimate PRIJE TRENINGA

	Ne konzumiram prije treninga	do 100 ml	od 100 do 200 ml	od 200 do 300 ml	od 300 do 500 ml	više od 500 ml
Voda						
Elektroliti						
Voćni sok						
Klasični cola napitci						
Zaslađena kava						
Zaslađeni čaj						
Energetski napitak (npr. Red Bull i sl.)						
Proteinski napitak						
Ugljikohidrati u tekućem obliku						
Aminokiseline						
Neki drugi napitak (ovdje odaberite količinu, a ispod navedite vrstu)						
Vrsta ______						

17. Označite koje su temperature tekućine koje pijete PRIJE TRENINGA (moguće je odabrati više odgovora)

	Svaki put	Povremeno	Rijetko	Nikada
Vrlo hladne (1 – 6°C)				
Hladne/Umjereno hladne (7–19°C)				
Sobne temperature (20-23°C)				
Tople/vruće (24 °C i više)				

18. Odaberite učestalost uzimanja tekućine TIJEKOM TRENINGA

	Svaki put	Povremeno	Rijetko	Nikada
Voda				
Elektroliti				
Voćni sok				
Klasični cola napitci				
Zaslađena kava				
Zaslađeni čaj				
Energetski napitak (npr. Red Bull i sl.)				
Proteinski napitak				
Ugljikohidrati u tekućem obliku				
Aminokiseline				
Nešto drugo (ovdje odaberite učestalost, a ispod navedite vrstu)				
Vrsta ________________________				

19. Odaberite količinu tekućine koju uzimate TIJEKOM TRENINGA

	Ne konzumiram tijekom treninga	do 100 ml	od 100 do 200 ml	od 200 do 300 ml	od 300 do 500 ml	više od 500 ml
Voda						
Elektroliti						
Voćni sok						
Klasični cola napitci						
Zaslađena kava						
Zaslađeni čaj						
Energetski napitak (npr. Red Bull i sl.)						
Proteinski napitak						
Ugljikohidrati u tekućem obliku						
Aminokiseline						
Neki drugi napitak (ovdje odaberite količinu, a ispod navedite vrstu)						
Vrsta ________________						

20. Označite koje su obično temperature tekućine koje pijete TIJEKOM TRENINGA.

	Svaki put	Povremeno	Rijetko	Nikada
Vrlo hladne (1–6°C)				
Hladne/Umjereno hladne (7–19°C)				
Sobne temperature (20–23°C)				
Tople/vruće (24°C i više)				

21. Odaberite učestalost uzimanja tekućina POSLIJE TRENINGA.

	Svaki put	Povremeno	Rijetko	Nikada
Voda				
Elektroliti				
Voćni sok				
Klasični cola napitci				

	Svaki put	Povremeno	Rijetko	Nikada
Zaslađena kava				
Zaslađeni čaj				
Energetski napitak (npr. Red Bull i sl.)				
Proteinski napitak				
Ugljikohidrati u tekućem obliku				
Aminokiseline				
Nešto drugo (ovdje odaberite učestalost, a ispod navedite vrstu)				
Vrsta ___________				

22. Odaberite količinu tekućine koju uzimate POSLIJE TRENINGA

	Ne konzumiram nakon treninga	do 100 ml	od 100 do 200 ml	od 200 do 300 ml	od 300 do 500 ml	više od 500 ml
Voda						
Elektroliti						
Voćni sok						
Klasični cola napitci						
Zaslađena kava						
Zaslađeni čaj						
Energetski napitak (npr. Red Bull i sl.)						
Proteinski napitak						
Ugljikohidrati u tekućem obliku						
Aminokiseline						
Neki drugi napitak (ovdje odaberite količinu, a ispod navedite vrstu)						
Vrsta _______						

23. U kojem vremenskom razdoblju POSLIJE TRENINGA popijete najviše tekućine?

a) do 25 minuta poslije treninga
b) od 30 minuta do 1:00 sat poslije treninga
c) od 1:00 do 2:00 sata poslije treninga
d) od 2:00 do 3:00 sata poslije treninga

24. Označite koje su obično temperature tekućine koje pijete POSLIJE TRENINGA

	Svaki put	Povremeno	Rijetko	Nikada
Vrlo hladne (1–6°C)				
Hladne/Umjereno hladne (7–19°C)				
Sobne temperature (20–23°C)				
Tople/vruće (24°C i više)				

25. Odaberite razlog zašto konzumirate odabrane tekućine

	Osjećaj žeđi	Sprječavanja dehidracije	Sprječavanja gubitka elektrolita	Sprječavanje gubitka/ izgradnje mišićne mase	Sprječavanje/ odgoda umora	Ne uzimam ovu tekućinu
Voda						

	Osjećaj žeđi	Sprječavanja dehidracije	Sprječavanja gubitka elektrolita	Sprječavanje gubitka/ izgradnje mišićne mase	Sprječavanje/ odgoda umora	Ne uzimam ovu tekućinu
Elektroliti						
Voćni sok						
Klasični cola napitci						
Zaslađena kava						
Zaslađeni čaj						
Energetski napitak (npr. Red Bull i sl.)						
Proteinski napitak						
Ugljikohidrati u tekućem obliku						
Aminokiseline						
Nešto drugo (ovdje odaberite razlog, a vrstu tekućine navedite ispod)						
Vrsta napitka ______________						

26. Odaberite u postocima koliko više tekućine unosite tijekom toplijeg dijela godine.

	0%	do 20%	od 20 do 50%	50 do 80%	više od 80%
Voda					
Elektroliti					
Voćni sok					
Klasični cola napitci					
Zaslađena kava					
Zaslađeni čaj					
Energetski napitak (npr. Red Bull i sl.)					
Proteinski napitak					
Ugljikohidrati u tekućem obliku					
Aminokiseline					
Nešto drugo (ovdje odaberite postotak, a ispod navedite vrstu)					
Vrsta napitka ___________					

27. Kako biste ocijenili svoju konzumaciju tekućine?

a) loše b) zadovoljavajuće c) dobro d) vrlo dobro e) izvrsno

28. Odaberite od koga i koliko često dobivate glavne informacije o hidraciji?

	Svaki put	Povremeno	Rijetko	Nikada
Trenera				
Kolega sportaša				
Liječnika				
Nutricionista				
Drugi izvor informacija (ovdje označite učestalost, a ispod upišite izvor)				

	Svaki put	Povremeno	Rijetko	Nikada
Drugi izvor informacija o hidraciji je ___________				
