# Supplementary material for: Self-Reported Changes in Exercise-Related Hydration Practices over a 30-Day Period in Physically Active University Students
Source: Sports (Basel). 2026 Sep 1;14(9):381. doi: 10.3390/sports14090381 (PMC13611659; doi:10.3390/sports14090381)
Supplement: Supplementary file 1 [file sports-14-00381-s001.zip › File_S2_English_Questionnaire.pdf]

FLUID INTAKE AMONG ATHLETES
English translation of the original Croatian questionnaire

Dear athletes,

We are conducting a survey on fluid intake among athletes involved in various sports activities. The survey is anonymous and is intended for athletes 18 years of age and older. The collected results will be used for scientific analysis. The questionnaire takes approximately 5 minutes to complete.
Thank you for your time and participation.

1) Country where you live._______________________________

2) The climate where you usually train.
a) Tropical climate b) Dry climate c) Temperate climate d) Snow-forest climate e) Polar (or snow) climate

3) Gender a) Male b) Female

4) Age

5) Height (cm) _________________

6. Weight (kg) ____________________

7) How would you describe your health status?
a) poor b) satisfactory c) good d) very good e) excellent

8) Which sport do you currently practice?___________________________

9) How many years have you practiced this sport? _________________________

10) Select the statement that best describes you as an athlete.
a) I do not participate in competitions
b) I compete at the regional level
c) I compete at the national level
d) I compete nationally and internationally, but I am not a member of the national team
e) I compete nationally and internationally and I am a member of the national team
f) I am an Olympian

11) What is the average number of training sessions you have per week?
a) 3–4 b) 5–7 c) 8–10 d) 11–14 e) 15 or more

12) What is the average number of hours you train per week?
a) 4 –5 b) 6–8 c) 9 – 12 d) 13–16 e) 17 or more

13) What training intensity predominates in your sessions?
a) intensity from 30% to 40% (low)
b) intensity from 40% to 50% (moderate)
c) intensity from 50% to 70% (medium)
d) intensity from 70% to 90% (submaximal)
e) intensity of 90% or higher (maximal)

14) How long PRIOR TO TRAINING do you drink the most fluids?
a) up to 25 minutes before training

b) from 30 minutes to 1 hour before training
c) 1 to 2 hours before training
d) 2 to 3 hours before training

15) What is the frequency of intake of the following fluids PRIOR TO TRAINING?

	Every time	Occasionally	Rarely	Never
Water				
Electrolyte beverages				
Fruit juice				
Classic cola drinks				
Sweetened coffee				
Sweetened tea				
Energy drinks (e.g. Red Bull)				
Protein drinks				
Liquid carbohydrate drinks				
Amino acids				
Other: (select frequency here and enter type below)				
Type				

16) How much of the following fluids do you usually drink PRIOR TO training?

	I do not consume this fluid prior to training	up to 100 mL	100–200 mL	200–300 mL	300–500 mL	more than 500 mL
Water						
Electrolyte beverages						
Fruit juice						
Classic cola drinks						
Sweetened coffee						
Sweetened tea						
Energy drinks (e.g. Red Bull)						
Protein drinks						
Liquid carbohydrate drinks						
Amino acids						
Other: (select amount here and enter type below)						
Type						

17) What is the temperature of the fluids you drink PRIOR TO TRAINING (you may select multiple answers)?

	Every time	Occasionally	Rarely	Never
Very cold (1–6°C)				
Cold/Quite cold (7–19°C)				
Room temperature (20–23°C)				
Warm/hot (24°C and above)				

18) How frequently do you drink fluids DURING TRAINING?

	Every time	Occasionally	Rarely	Never
Water				
Electrolyte beverages				
Fruit juice				

	Every time	Occasionally	Rarely	Never
Classic cola drinks				
Sweetened coffee				
Sweetened tea				
Energy drinks (e.g. Red Bull)				
Protein drinks				
Liquid carbohydrate drinks				
Amino acids				
Other: (select frequency here and enter type below)				
Type				

19) How much of the following fluids do you usually drink DURING TRAINING?

	I do not consume this fluid during training	up to 100 mL	100–200 mL	200–300 mL	300–500 mL	more than 500 mL
Water						
Electrolyte beverages						
Fruit juice						
Classic cola drinks						
Sweetened coffee						
Sweetened tea						
Energy drinks (e.g. Red Bull)						
Protein drinks						
Liquid carbohydrate drinks						
Amino acids						
Other: (select amount here and enter type below)						
Type						

20) What is the temperature of the fluids you drink DURING TRAINING (you may select multiple answers).

	Every time	Occasionally	Rarely	Never
Very cold (1–6°C)				
Cold/Quite cold (7–19°C)				
Room temperature (20–23°C)				
Warm/hot (24°C and above)				

21) How frequently do you drink fluids AFTER TRAINING?

	Every time	Occasionally	Rarely	Never
Water				
Electrolyte beverages				
Fruit juice				
Classic cola drinks				
Sweetened coffee				
Sweetened tea				
Energy drinks (e.g. Red Bull)				
Protein drinks				
Liquid carbohydrate drinks				
Amino acids				
Other: (select frequency here and enter type below)				
Type				

22) How much of the following fluids do you usually drink AFTER TRAINING?

	I do not consume this fluid after training	up to 100 mL	100–200 mL	200–300 mL	300–500 mL	more than 500 mL
Water						
Electrolyte beverages						
Fruit juice						
Classic cola drinks						
Sweetened coffee						
Sweetened tea						
Energy drinks (e.g. Red Bull)						
Protein drinks						
Liquid carbohydrate drinks						
Amino acids						
Other: (select amount here and enter type below)						
Type						

23) When do you ingest the largest quantity of fluids AFTER TRAINING?

a) up to 25 minutes after training
b) 30 minutes to 1 hour after training
c) 1 to 2 hours after training
d) 2 to 3 hours after training

24. What is the temperature of the fluids you drink AFTER TRAINING (you may select multiple answers).

	Every time	Occasionally	Rarely	Never
Very cold (1–6°C)				
Cold/Quite cold (7–19°C)				
Room temperature (20–23°C)				
Warm/hot (24°C and above)				

25. What is the main reason you consume each of the following beverages?

	Thirst	Preventing dehydration	Preventing loss of electrolytes	Preventing muscle mass loss and building muscle mass	Preventing or postponing fatigue	I do not drink this liquid
Water						
Electrolyte beverages						
Fruit juice						
Classic cola drinks						
Sweetened coffee						
Sweetened tea						
Energy drink (e.g. Red Bull)						
Protein drink						
Liquid carbohydrate drinks						

	Thirst	Preventing dehydration	Preventing loss of electrolytes	Preventing muscle mass loss and building muscle mass	Preventing or postponing fatigue	I do not drink this liquid
Amino acids						
Other: (select reason here and enter type below)						
Type						

26. During warmer seasons, how much do you increase your intake of these fluids?

	0%	up to 20%	from 20 to 50%	from 50 to 80%	more than 80%
Water					
Electrolyte beverages					
Fruit juice					
Classic cola drinks					
Sweetened coffee					
Sweetened tea					
Energy drinks (e.g. Red Bull)					
Protein drinks					
Liquid carbohydrate drinks					
Amino acids					
Other: (select percentage here and enter type below)					
Type					

27. How would you assess your overall fluid intake?

a) poor b) satisfactory c) good d) very good e) excellent

28. How often do you receive information about hydration, and from whom?

	Every time	Occasionally	Rarely	Never
Coach				
Fellow athletes				
Physician				
Nutritionist				
Other: (select frequency here and enter source below)				
Source				
