# Supplementary material for: Self-Reported Changes in Exercise-Related Hydration Practices over a 30-Day Period in Physically Active University Students
Source: Sports (Basel). 2026 Sep 1;14(9):381. doi: 10.3390/sports14090381 (PMC13611659; doi:10.3390/sports14090381)
Supplement: Supplementary file 1 [file sports-14-00381-s001.zip › Supplementary_Table_S1_Questionnaire_Items_and_Use.pdf]

Supplementary Table S1

Questionnaire items and their use in the present study

The complete questionnaire contains 28 questions. The test–retest agreement analysis focused on selected beverage-consumption frequency items, while other items were used for participant characterization, descriptive presentation, or were not included in the present analysis.

Question(s)	Content	Use in the present study	Location or note
1–2	Country of residence and usual training climate	Not reported in the present manuscript	Background questionnaire items
3–6	Sex, age, height, and body mass	Participant characterization	Tables 1 and 2; BMI calculated from self-reported height and body mass
7	Self-rated health status	Not reported in the present manuscript	Collected but not included in the current analyses
8	Sport practiced	Participant characterization	Section 2.1 (Participants)
9	Years of sport participation	Not reported in the present manuscript	Collected but not included in the current analyses
10–12	Competitive status, weekly training frequency, and weekly training duration	Participant characterization	Tables 1 and 2
13	Predominant training intensity	Not reported in the present manuscript	Collected but not included in the current analyses
14 and 23	Timing of greatest fluid intake before and after training	Descriptive analysis based on the first administration	Figure 1
15, 18, and 21	Frequency of beverage consumption before, during, and after training	Primary item-level test–retest agreement analysis	Table 3; sweetened coffee, sweetened tea, and other beverages were excluded because paired test–retest responses were unavailable
16, 19, and 22	Amounts of beverages consumed before, during, and after training	Descriptive analysis based on the first administration	Table 4
17, 20, and 24	Temperature of beverages before, during, and after training	Not reported in the present manuscript	Not included in the current analyses
25	Reasons for beverage consumption	Not reported in the present manuscript	Not included in the current analyses
26	Seasonal increase in beverage intake	Not reported in the present manuscript	Not included in the current analyses
27	Self-assessment of overall fluid intake	Not reported in the present manuscript	Not included in the current analyses
28	Sources and frequency of hydration-related information	Descriptive analysis based on the first administration	Figure 2

Note. Questions 15, 18, and 21 are matrix questions containing separate ordinal responses for each beverage category. The supplementary questionnaires reproduce all items and response options. Table 3 is based on paired responses from both administrations, whereas Table 4 and Figures 1–2 present data from the first administration.
